# Complete Genome Sequence of a Bovine Ephemeral Fever Virus Isolate from Israel

**DOI:** 10.1128/MRA.00822-19

**Published:** 2019-10-10

**Authors:** Daniel L. W. Dorey-Robinson, Mar Fernández de Marco, Luis M. Hernández-Triana, Arran J. Folly, Lorraine M. McElhinney, Jessica E. Stokes, Chris Sanders, Simon Carpenter, Anthony R. Fooks, Olga Zalesky, Boris Gelman, Oran Erster, Nicholas Johnson

**Affiliations:** a Animal and Plant Health Agency, Addlestone, Surrey, United Kingdom; b The Pirbright Institute, Woking, Surrey, United Kingdom; c The Kimron Veterinary Institute, Bet Dagon, Israel; DOE Joint Genome Institute

## Abstract

Here, we report the first complete genome of a bovine ephemeral fever virus (BEFV) isolate from an infected bovine in Israel. The genome shares 95.3% identity with a Turkish genomic sequence but contains α3 and γ open reading frames that are truncated compared to those of existing BEFV genome sequences.

## ANNOUNCEMENT

Bovine ephemeral fever virus (BEFV) is the causative agent of bovine ephemeral fever (BEF) in cattle. BEFV is a member of the family Rhabdoviridae and the genus Ephemerovirus. The virus has a single-stranded negative-sense genome approximately 15 kb in length and contains open reading frames (ORFs) coding for a nucleoprotein (N), phosphoprotein (P), matrix protein (M), glycoprotein (G), second glycoprotein (GNS), five short ORFs (α1, α2, α3, β, and γ), and an RNA-dependent RNA polymerase (L) (1–3).

BEFV is divided into three geographically defined phylogenetic clusters from Australia, the Middle East, and East Asia (4, 5). However, no genome representation exists for African BEFVs, despite repeated detection of the virus across sub-Saharan Africa (3). Currently, there are only six publicly accessible complete BEFV genomes. Here, we report the first complete genome sequence from a virus isolated, in Vero cells, from a cow (Bos taurus) in Israel in 2006.

Total RNA was extracted from infected Vero cells using TRIzol (Invitrogen) and reverse transcribed using a cDNA synthesis kit (Roche) before purification using AMPure XP beads (Beckman Coulter). The Nextera XT DNA library preparation kit (2 × 150-bp reads; Illumina) was used for library preparation. Sequencing was carried out on an Illumina MiSeq sequencer, which generated 31,922,946 reads in total. The reads were aligned and genome completeness assessed against the Turkish full-genome sequence (GenBank accession number KY012742), since both sequences were likely to be of the Middle Eastern phylogroup. An in-house script utilizing Burrows-Wheeler Aligner (BWA) v0.7.13 (6) and SAMtools v1.9 was used to iteratively construct the consensus sequence, with visual inspection using Tablet (7). The resulting contig consisted of 5,050,961 reads (15.82% of total), with an average depth of 42,614× and a maximum depth of 121,098×.

The BEFV genome from Israel is 14,959 nucleotides long with 33.73% GC content and contains the expected BEFV ORFs. Lasergene MegAlign v14.1.0 was used to evaluate nucleotide similarity. The Israeli sequence shared 95.3% identity with its closest relative (GenBank accession number KY012742) and between 90.0% and 91.6% identity to sequences from Australia and East Asia. Identification of ORFs was based on annotations from other BEFV records from GenBank. All ORFs were identical in length compared to the complete genomes from Australia (AF234533) and Turkey (KY012742), with the exception of α3, β, and γ ORFs. The α3 coding sequence contained a stop codon resulting from a C-to-T substitution at position 7268, which produced a shorter ORF. The β ORF was 49 residues longer than that of the Australian genome (AF234533) but the same length as that of the Turkish genome (KY012742). The γ ORF contained a population change at nucleotide 8162 from a G (4,483 reads) to an A (6,312 reads), which introduced a stop codon. No function has been assigned to the α3, β, or γ ORF, and similar changes during cell culture have been reported (3). In addition, we noted a reduced average read depth over a 2,598-bp sequence covering the GNS and five accessory proteins. This could indicate the presence of defective interfering particles that have previously been reported for BEFV (8), which could have developed during tissue culture passaging.

### Data availability.

The full-genome sequence has been deposited in GenBank under the accession number MN078236, and raw data have been deposited in the European Nucleotide Archive under the accession number ERR3506784. The script used to generate the data is available at https://github.com/Daniel-DoreyRobinson/Bioinformatics/blob/master/refguidealignb.sh.
